# Supplementary material for: Escherichia coli Isolated from Cases of Colibacillosis in Russian Poultry Farms (Perm Krai): Sensitivity to Antibiotics and Bacteriocins
Source: Microorganisms. 2020 May 15;8(5):741. doi: 10.3390/microorganisms8050741 (PMC7285186; doi:10.3390/microorganisms8050741)
Supplement: Supplementary file 1 [file microorganisms-08-00741-s001.pdf]

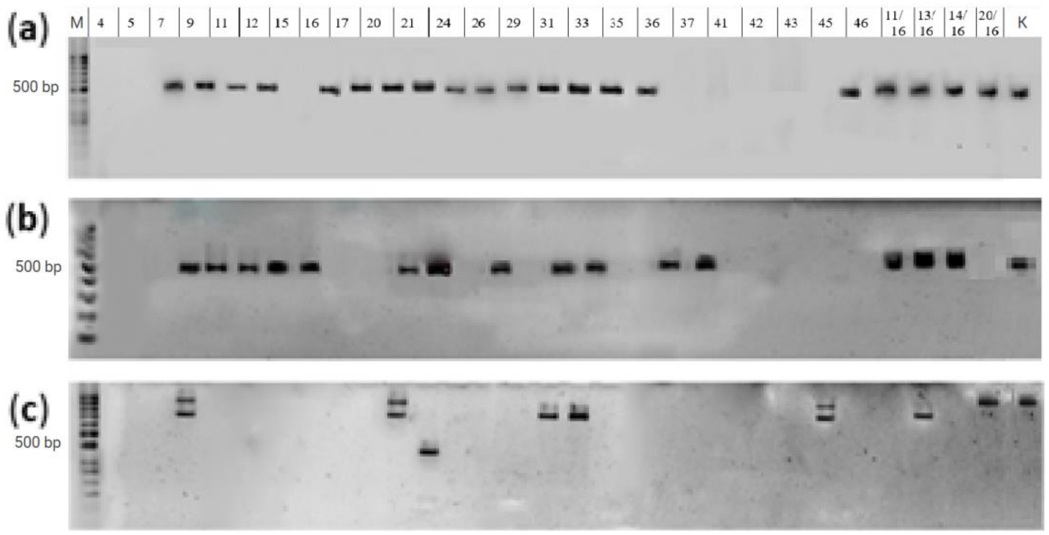

Figure S1. Electrophoregrams of PCR products: (**a**) amplification of bla_{TEM}, (**b**) $bla_{\text{CTX-M}}$ and (**c**) class 1 integron fragments. Legend: M - molecular mass marker GeneRuler 100 bp Plus DNA Ladder, 4-20|16 - numbers of examined APEC strains, K - positive isolate for each gene (controle PCR-reaction).

Table S1. Primers for PCR-detection of ESBL-genes and class 1 integrons.

Target	Primer Name	Nucleotide Sequence	Product Size (bp)	Reference
bla_{TEM}	TEM-C	5′-ATCAGCAATAAACCAGC-3′	516	[1]
	TEM-H	5′-CCCCGAAGAACGTTTTC-3′		
bla_{SHV}	SHV-F	5′-AGGATTGACTGCCTTTTTG-3′	392	[1]
	SHV-R	5′-ATTTGCTGATTTCGCTCG-3′		
bla_{OXA}	OXA-F	5′-ATATCTCTACTGTTGCATCTCC-3′	619	[1]
	OXA-R	5′-AAACCCTTCAAACCATCC-3′		
$bla_{\text{CTX-M}}$	CTX-M-F	5′-CGCTTTGCGATGTGCAG-3′	551	[2]
	CTX-M-R	5′-ACCGCGATATCGTTGGT-3′		
bla_{CMY}	CMY-2-F	5′-GCAGGCYATTCCGGGTATG-3′	915	[3]
	CMY-2-R	5′-GCYACGTAGCTGCCAAAYCC-3′		
Integrons	5′CS	5′-GGCATCCAAGCAGCAAG-3′	-*	[4]
	3′CS	5′-AAGCAGACTTGACCTGA-3′		

*- the product is represented by sizes of different lengths.

References:

1. Aleisa, A.M.; Ashgan, M.H.; Alnasserallah, A.A.; Mahmoud, M.H.; Moussa, I. M. Molecular detection of β-lactamases and aminoglycoside resistance genes among *Escherichia coli* isolates recovered from medicinal plant 2013. *Afr. J. Microbiol. Res*. **2013**, 7(20), 2305-2310. [CrossRef]
2. Ahmed, A.M.; Motoi, Y.; Sato, M.; Maruyama, A.; Watanabe, H.; Fukumoto, Y.; Shimamoto, T. Zoo animals as reservoirs of gram-negative bacteria harboring integrons and antimicrobial resistance genes. *Appl. Environ. Microbiol*. **2007**, 73(20), 6686-6690. [CrossRef] [PubMed]
3. Koo, H.J.; Woo, G.J. Characterization of antimicrobial resistance of *Escherichia coli* recovered from foods of animal and fish origin in Korea. *J. Food Prot*. **2012**, 75(5), 966-972. [CrossRef] [PubMed]
4. Lévesque, C.; Piché, L.; Larose, C.; Roy, P.H. PCR mapping of integrons reveals several novel combinations of resistance genes. *Antimicrob. Agents Chemother*. **1995**, 39(1), 185-191. [CrossRef] [PubMed]
